# Awareness of human papillomavirus and acceptability of the vaccine among women in Palestine: is it time for policy adjustment?

**DOI:** 10.1186/s12905-022-01930-8

**Published:** 2022-08-19

**Authors:** Mohamedraed Elshami, Hanan Abukmail, Ibrahim Al-Slaibi, Mohammed Alser, Afnan Radaydeh, Alaa Alfuqaha, Mariam Thalji, Salma Khader, Lana Khatib, Nour Fannoun, Bisan Ahmad, Lina Kassab, Hiba Khrishi, Deniz Houssaini, Nour Abed, Aya Nammari, Tumodir Abdallah, Zaina Alqudwa, Shahd Idais, Ghaid Tanbouz, Ma’alem Hajajreh, Hala Abu Selmiyh, Zakia Abo-Hajouj, Haya Hebi, Manar Zamel, Refqa Najeeb Skaik, Lama Hammoud, Saba Rjoub, Hadeel Ayesh, Toqa Rjoub, Rawan Zakout, Amany Alser, Nasser Abu-El-Noor, Bettina Bottcher

**Affiliations:** 1 Division of Surgical Oncology, Department of Surgery, University Hospitals Cleveland Medical Center, 11100 Euclid Avenue, Lakeside 7100, Cleveland, OH 44106 USA; 2 Ministry of Health, Gaza, Palestine; 3 Faculty of Medicine, Islamic University of Gaza, Gaza, Palestine; 4 Almakassed Hospital, Jerusalem, Palestine; 5 Faculty of Medicine, Al-Quds University, Jerusalem, Palestine; 6 Faculty of Graduate Studies, An-Najah National University, Nablus, Palestine; 7 Faculty of Medicine, An-Najah National University, Nablus, Palestine; 8 Faculty of Pharmacy, Alazhar University of Gaza, Gaza, Palestine; 9 Faculty of Dentistry and Dental Surgery, Al-Quds University, Jerusalem, Palestine; 10 Faculty of Medicine, Alazhar University of Gaza, Gaza, Palestine; 11 Hebron Governmental Hospital, Hebron, Palestine; 12 Alia Hospital, Hebron, Palestine; 13 Al-Shiffa Hospital, Gaza, Palestine; 14 Faculty of Nursing, Islamic University of Gaza, Gaza, Palestine

**Keywords:** Human papillomavirus, Cervical cancer, Vaccine, Vaccination, Health education, Awareness, Palestine

## Abstract

**Background:**

Progress has been made in the reduction of morbidity and mortality of cervical cancer by the implementation of human papillomavirus (HPV) vaccination programs. This study aimed to assess the awareness of Palestinian women about HPV as well as their knowledge and acceptability of the HPV vaccine and to examine the factors associated with good awareness.

**Methods:**

This was a national cross-sectional study. Adult women were recruited from hospitals, primary healthcare centers, and public spaces in 11 Palestinian governorates using convenience sampling. A structured questionnaire was used for data collection. For each correctly answered question, one point was given. The total score was calculated and categorized into poor (0–10) and good awareness (11–21).

**Results:**

The questionnaire was completed by 7223 women out of 8086 who were approached (response rate = 89.3%). A total of 7058 questionnaires were included in the final analysis; 4403 from the West Bank and Jerusalem (WBJ) and 2655 from the Gaza Strip. Women recruited from the Gaza Strip were younger, getting lower monthly incomes, and with fewer chronic diseases than women recruited from the WBJ.

Only 33 women (0.5%) displayed good awareness of HPV and its vaccine with 0.7% of women from WBJ and only 0.2% of women from the Gaza Strip. Completing post-secondary education, being employed or a student, and having a higher monthly income were associated with an increase in the likelihood of having good awareness.

Among women who had heard of HPV (n = 571, 8.1%), only 46 women (8.1%) reported familiarity with its vaccine. Women from the WBJ were more likely than women from the Gaza Strip to have heard about the HPV vaccine (0.9% vs. 0.2%). Most women agreed to receive the HPV vaccine themselves or for their daughters if it was given without cost or with a co-payment. No differences were found in the likelihood of agreeing to receive the HPV vaccine among women in the WBJ versus the Gaza Strip.

**Conclusion:**

The overall awareness of HPV and its vaccine was extremely low. Inclusion of the HPV vaccine in the national immunization program could change this, especially as the HPV vaccine appeared to be acceptable.

## Introduction

Cervical Cancer (CC) is a major health problem among women with more than 300,000 deaths reported globally in 2020 [1]. The burden of CC is substantially higher in low- and middle-income countries, where nine out of 10 women with CC die [2, 3]. However, CC is considered one of the most preventable and treatable types of cancer if detected early and managed appropriately [4].

Human papillomavirus (HPV) infection has been identified as the most significant risk factor [5–8]. A previous study conducted in the Gulf Cooperation Council countries showed that the high-risk HPV genotypes 16 and 18 were found in 21.0% of women tested. Women in Qatar (31.3%) had the highest positivity rate, followed by women in Bahrain (20.0%), Saudi Arabia (17.2%), and the United Arab Emirates (14.7%) [9]. In Palestine, HPV infection was detected in 25% of non-pregnant women, 68% of pregnant women, and 89% of CC specimens [10]. Women’s awareness about HPV infection as a risk factor for CC is crucial to facilitate engagement in cervical screening programs as well as acceptance of vaccination, particularly as HPV immunization programs have successfully reduced morbidity and mortality associated with CC [11, 12].

Vaccination against HPV is one of the primary prevention measures for CC [13]. Previous reports demonstrated this by finding 690,000 CC cases and 420,000 deaths prevented, mostly in low- and middle-income countries, by HPV vaccination of a cohort of 58 million 12-year-old girls in 179 countries [14]. HPV vaccination was found to be cost-effective in 156 (87%) of 179 countries included in the Papillomavirus Rapid Interface for Modelling and Economics [14]. Beside the preventive role of HPV vaccines, there has been a growing interest to extrapolate the clinical application of these vaccines to treat HPV infections and preinvasive HPV diseases. However, these vaccines are still under investigation and have not been introduced in clinical practice [15].

The HPV vaccine is not part of the national vaccination program in Palestine [16]. Understanding Palestinian women’s awareness, attitudes, and acceptance of the HPV vaccine could be of value for healthcare decision makers in proposing future policies to include the vaccine in the national program especially as no CC screening programs exist in Palestine [16].

This national study aimed to: (1) assess Palestinian women’s awareness of HPV and its vaccine, (2) examine if there is a difference in awareness between women from the Gaza Strip versus the West Bank and Jerusalem (WBJ), and (3) identify the factors associated with good awareness.

## Materials and methods

### Study design, setting and target population

A cross-sectional study was conducted from July 2019 to March 2020 in the two main areas in Palestine: the Gaza Strip and the WBJ. About half of the female population in Palestine is of reproductive age (15–49 years) [17]. Therefore, adult women (aged ≥ 18 years) were the target population. Eligible women were recruited from governmental hospitals, primary healthcare centers, and public spaces. The public spaces included malls, markets, parks, restaurants, mosques, churches, and transportation stations. Exclusion criteria included women holding citizenship other than Palestinian, working or studying in a health-related field, and visiting oncology departments or clinics.

### Sampling methods

Eligible women were recruited from hospitals, primary healthcare centers, and public spaces in 11 Palestinian governorates using convenience sampling. The data collection sites were located across Palestine and covered widespread geographical areas to create a study cohort that resembled the Palestinian community [18–20].

### Questionnaire and data collection

A structured questionnaire was designed using questions from previous similar studies [21–32]. A back-to-back translation was done, where two bilingual healthcare professionals translated the questionnaire from English to Arabic, and then the Arabic version was back-translated to English by another two bilingual healthcare professionals. Those healthcare professionals had relevant clinical and research experiences in gynecologic oncology, public health, and survey design. Then, different health experts and researchers assessed the content validity of the questionnaire. This was followed by conducting a pilot study (n = 130) to test the clarity of questions of the Arabic version of the questionnaire. Questionnaires from the pilot study were not included in the final analysis.

The questionnaire consisted of three sections. The first section described sociodemographic data including age, menarche, highest level of education, occupation, monthly income, marital status, knowing someone with cancer, place of residency, and having a chronic disease. The second section evaluated the awareness of HPV. The third section assessed the awareness and acceptability of the HPV vaccine.

Yes/no questions were used to assess the awareness of HPV including its transmission and relation to developing any type of cancer. The participant’s knowledge of the availability of the HPV vaccine in the national vaccination program as well as their thoughts if the HPV vaccine could help in protecting against CC was assessed using yes/no/do not know questions. The open-ended recall questions were posed to specify the relationship of HPV with any cancer types, the age at which the HPV vaccine should be first given, and the number of HPV vaccine doses needed. The participants’ responses were then converted to correct/incorrect responses based on the Centers for Disease Control and Prevention information [33, 34]. A 5-point Likert scale (1 = strongly disagree, 5 = strongly agree) was used to evaluate the acceptability of the receipt of the HPV vaccine among study participants who had previously heard about it.

Eligible women were invited to complete the questionnaire in a face-to-face interview. Data collection was done utilizing the ‘Kobo Toolbox’, a secure, user-friendly tool that can be accessed by smartphones [35]. A special training had been run for all data collectors, who were all female and had a medical background, on how to use Kobo Toolbox, recruit participants, and facilitate the completion of the questionnaire.

### Statistical analysis

Descriptive statistics were used to summarize participants’ characteristics. Continuous, non-normally distributed variables were summarized using the median [interquartile range (IQR)] with baseline comparisons performed using Kruskal–Wallis tests. Categorical variables were summarized using frequencies (n) and percentages (%) with baseline comparisons performed using Fisher’s exact test. Since 1450 NIS (about $450) was the minimum wage of Palestinian employees [36], it was chosen to categorize monthly income into two categories: < 1450 and ≥ 1450 NIS.

To evaluate women’s awareness of HPV and its vaccine, a scoring system was adapted from previous studies [18, 37–40]. Each correct answer was given one point. The total score (ranging from 0 to 21) was calculated and categorized into two categories: poor (0–10) and good awareness (11–21). The awareness of HPV and its vaccine was compared between women from the Gaza Strip versus the WBJ using Fisher’s exact test. The 5-point Likert scale was converted into a 3-point scale to facilitate comparisons especially as it was difficult for participants to differentiate between ‘agree’ versus ‘strongly agree’ and ‘disagree’ versus ‘strongly disagree’. Therefore, the responses of ‘strongly agree’ and ‘strongly disagree’ were recoded to ‘agree’ and ‘disagree’ respectively.

Missing data occurred completely at random. Therefore, a complete case analysis was utilized to handle missing data. Data were analyzed using Stata software version 16.0 (StataCorp, College Station, Texas, United States).

## Results

The questionnaire was completed by 7223 women out of 8086 who were approached (response rate = 89.3%). In total, 7058 questionnaires were included in the analysis (30 did not meet the inclusion criteria, 135 had missing data); 4403 from the WBJ and 2655 from the Gaza Strip.

The median age [IQR] for all women included in the study was 32.0 years [24.0, 42.0] (Table 1). Women recruited from the Gaza Strip were younger, getting lower monthly income, and with less chronic diseases than women recruited from the WBJ.

**Table 1:** Characteristics of study participants

Characteristic	Total (n = 7058)	Gaza Strip (n = 2655)	WBJ (n = 4403)	*p*-value
*Age*, median [IQR]	32 [24, 42]	30 [24, 39]	33 [24, 44]	< 0.001
*Age group*, n (%)				
18–20	756 (10.7)	249 (9.4)	507 (11.5)	< 0.001
21–40	4331 (61.4)	1809 (68.1)	2522 (57.3)	
41 or older	1971 (27.9)	597 (22.5)	1374 (31.2)	
*Educational level*, n (%)				
Secondary or below	3893 (55.2)	1497 (56.4)	2396 (54.4)	0.11
Above secondary	3165 (44.8)	1158 (43.6)	2007 (45.6)	
*Occupation*, n (%)				
Housewife	4647 (65.8)	2008 (75.6)	2639 (59.9)	< 0.001
Employed	1476 (20.9)	348 (13.1)	1128 (25.6)	
Retired	69 (1.0)	11 (0.5)	58 (1.4)	
Student	866 (12.3)	288 (10.8)	578 (13.1)	
*Monthly income* ≥ *1450 NIS*, n (%)	4666 (66.1)	693 (26.1)	3973 (90.2)	< 0.001
*Marital status*, n (%)				
Single	1657 (23.5)	527 (19.8)	1130 (25.7)	< 0.001
Married	5058 (71.6)	2025 (76.3)	3033 (68.8)	
Divorced/Widowed	343 (4.9)	103 (3.9)	240 (5.5)	
*Having a chronic disease*, n (%)	1397 (19.8)	417 (15.7)	980 (22.3)	< 0.001
*Knowing someone with cancer*, n (%)	4083 (57.9)	1483 (55.9)	2600 (59.1)	0.009
*Site of data collection*, n (%)				
Public spaces	2.695 (38.2)	863 (32.5)	1832 (41.6)	< 0.001
Hospitals	1890 (26.7)	642 (24.2)	1248 (28.4)	
Primary healthcare centers	2473 (35.1)	1150 (43.3)	1323 (30.0)	

n, number of participants; IQR, interquartile range; WBJ, West Bank and Jerusalem

### Good awareness and its associated factors

Only 33 women (0.5%) displayed good awareness of HPV and its vaccine, defined as having a score of 11–21 (Table 2). Women living in the WBJ were 3.5 times more likely than women living in the Gaza Strip to have good awareness (0.7% vs. 0.2%).

**Table 2:** Awareness level of human papillomavirus and its vaccine among study participants

Level	Total (n = 7058)	Gaza Strip (n = 2655)	WBJ (n = 4403)	*p*-value
Poor (total score ranged from 1 to 10)	7025 (99.5)	2651 (99.8)	4374 (99.3)	0.002
Good (total score ranged from 11 to 21)	33 (0.5)	4 (0.2)	29 (0.7)	

n, number of participants; WBJ, West Bank and Jerusalem

Data are presented as frequencies and percentages

Besides living in the WBJ, completing post-secondary education, being employed or a student, and having a higher monthly income were all associated with higher likelihood to have good awareness (Table 3). On the other hand, visiting governmental hospitals or primary healthcare centers was associated with lower likelihood to have good awareness.

**Table 3:** Association between having a good awareness of human papillomavirus and its vaccine with participant characteristics

	Poor awareness (n = 7025)	Good awareness (n = 33)	*p*-value
*Age group*			
18–20	750 (10.7)	6 (18.2)	0.06
21–40	4308 (61.3)	23 (69.7)	
41 or older	1967 (28.0)	4 (12.1)	
*Educational level*			
Secondary or below	3883 (55.3)	10 (30.3)	0.005
Above secondary	3142 (44.7)	23 (69.7)	
*Occupation*			
Housewife	4635 (66.0)	12 (36.4)	0.003
Employed	1464 (20.8)	12 (36.4)	
Retired	69 (1.0)	0	
Student	857 (12.2)	9 (27.2)	
*Monthly income*			
< 1450 NIS	2389 (34.0)	3 (9.1)	0.001
≥ 1450 NIS	4636 (66.0)	30 (90.9)	
*Marital status*			
Single	1645 (23.4)	12 (36.4)	0.06
Married	5040 (71.7)	18 (54.5)	
Divorced/Widowed	340 (4.9)	3 (9.1)	
*Residency*			
Gaza Strip	2651 (37.7)	4 (12.1)	0.002
WBJ	4374 (62.3)	29 (87.9)	
*Having a chronic disease*			
No	5632 (80.2)	29 (87.9)	0.38
Yes	1393 (19.8)	4 (12.1)	
*Knowing someone with cancer*			
No	2961 (42.1)	14 (42.4)	0.99
Yes	4064 (57.9)	19 (57.6)	
*Site of data collection*			
Public spaces	2673 (38.0)	22 (66.7)	0.002
Hospitals	1883 (26.9)	7 (21.2)	
Primary healthcare centers	2469 (35.1)	4 (12.1)	

n, number of participants; WBJ, West Bank and Jerusalem

### Awareness of HPV

Out of the 7058 women included in the study, only 571 (8.1%) had heard about HPV; 339 of 4403 in the WBJ (7.7%) and 232 of 2655 women in the Gaza Strip (8.7%). When those 571 women were asked about the mode of transmission of HPV, 95 (16.6%) and 53 (9.3%) answered correctly with ‘sexually’ and ‘mother to newborn’, respectively (Table 4). Women from the WBJ were more likely than women from the Gaza Strip to give correct answers about the HPV mode of transmission.

**Table 4:** Awareness of human papillomavirus among study participants who heard of it

Question	Total (n = 571)	Gaza Strip (n = 232)	WBJ (n = 339)	*p*-value
*Mode of transmission*				
Airborne infection	29 (5.1)	12 (5.2)	17 (5.0)	0.99
Blood transfusion	116 (20.3)	39 (16.8)	77 (22.7)	0.09
Contaminated food	16 (2.8)	10 (4.3)	6 (1.8)	0.12
Sexually	95 (16.6)	26 (11.2)	69 (20.4)	0.004
Mother to newborn	53 (9.3)	10 (4.3)	43 (12.7)	< 0.001
Others	17 (2.9)	8 (3.4)	9 (2.7)	0.62
*HPV can infect*				
Males	9 (1.6)	3 (1.3)	6 (1.8)	0.93
Females	141 (24.7)	57 (24.6)	84 (24.8)	
Both	355 (62.1)	147 (63.3)	208 (61.3)	
Do not know	66 (11.6)	25 (10.8)	41 (12.1)	
*Heard about any relation between HPV and any type of cancer*	214 (37.5)	77 (33.2)	137 (40.4)	0.09
*Type of cancer caused by HPV*^#^				
Cervical cancer	107 (50.0)	23 (29.9)	84 (61.3)	< 0.001
Skin cancer	3 (1.4)	0	3 (2.2)	0.55
Penile cancer	2 (0.9)	0	2 (1.5)	0.54
Incorrect answers	89 (41.6)	49 (63.6)	40 (29.2)	< 0.001
Do not know	25 (11.7)	8 (10.5)	17 (12.4)	0.83

n, number of participants; WBJ, West Bank and Jerusalem; HPV, human papillomavirus

^**#**^The denominator is the number of participants who recognized a relation between human papillomavirus and any type of cancer

Data are presented as frequencies and percentages

About two-thirds (n = 355, 62.1%) correctly answered that HPV can infect both males and females. About one-third (n = 214, 37.5%) had heard about a relationship between HPV and any type of cancer. Of those, 107 (50.0%) identified CC, 3 (1.4%) identified skin cancer and 2 (0.9%) identified penile cancer. Women from the Gaza Strip were more likely than women from the WBJ to give incorrect answers about HPV-related cancers.

### Awareness and acceptability of HPV vaccine

Among women who had heard of HPV (n = 571), only 46 (8.1%) had also heard of the HPV vaccine: 40 in the WBJ (87.0%) and 6 in the Gaza Strip (13.0%). Women from the WBJ were more likely than women from the Gaza Strip to have heard about the HPV vaccine (0.9% vs. 0.2%).

Women who had heard of the HPV vaccine (n = 46) were asked some questions about it. No differences in the likelihood to give correct answers to these questions were found between women in the WBJ versus the Gaza Strip. When asked about the age at which the HPV vaccine is first given, 19 women (41.3%) answered correctly with a range from 9 to 14 years old (Table 5). In addition, 24 (52.1%) gave a correct answer for the number of HPV vaccine doses that should be taken (i.e., two or three doses). More than half of the women who had heard of the HPV vaccine (n = 25, 54.3%) correctly answered that the HPV vaccine can be given to both males and females. However, about one-third (n = 15, 32.6%) claimed that the HPV vaccine is part of the Palestinian Ministry of Health vaccination program, and 17 women (37.0%) did not know if the vaccine was part of the program or not. The vast majority of women who had heard of the HPV vaccine stated that it could help to protect against CC (n = 44, 95.6%).

**Table 5:** Awareness of human papillomavirus vaccine among study participants who heard about it

Question	Total (n = 46)	Gaza Strip (n = 6)	WBJ (n = 40)	*p*-value
*Age at which HPV vaccine is first given*				
Correct answers	19 (41.3)	1 (17.0)	18 (45.0)	0.38
Incorrect answers	27 (58.7)	5 (83.0)	22 (55.0)	
*Number of HPV vaccine doses that should be taken*				
Correct answers	24 (52.1)	4 (67.0)	20 (50.0)	0.67
Incorrect answers	22 (47.9)	2 (33.0)	20 (50.0)	
*HPV vaccine can be given to*				
Males	0	0	0	0.05
Females	19 (41.3)	0	19 (48.0)	
Both	25 (54.3)	6 (100.0)	19 (48.0)	
Do not know	2 (4.4)	0	2 (4.0)	
*HPV vaccine is a part of the Palestinian Ministry of Health vaccination program*				
No	14 (30.4)	4 (66.0)	10 (25.0)	0.22
Yes	15 (32.6)	1 (17.0)	14 (35.0)	
Do not know	17 (37.0)	1 (17.0)	16 (40.0)	
*HPV vaccine can help to protect against cervical cancer*				
No	2 (4.4)	0	2 (5.0)	0.99
Yes	44 (95.6)	6 (100.0)	38 (95.0)	
Do not know	0	0	0	

n, number of participants; WBJ, West Bank and Jerusalem; HPV, human papillomavirus

Data are presented as frequencies and percentages

No differences were found in the likelihood of agreeing to receive the HPV vaccine among women, who had heard about the vaccine, in the WBJ versus the Gaza Strip (Table 6). Forty-one women (89.1%) would agree to receive the HPV vaccine if it was given for free and 39 (84.8%) if they had to pay for it. In addition, 42 (91.3%) would agree for their daughters to receive the HPV vaccine, and 40 (87.0%) also if it incurred a cost.

**Table 6:** Acceptability for the receipt of human papillomavirus vaccine among study participants who heard of it

Question	Total (n = 46)			Gaza Strip (n = 6)			WBJ (n = 40)			*p*-value
	Disagree	Not sure	Agree	Disagree	Not sure	Agree	Disagree	Not sure	Agree	
Would you like to receive the HPV vaccine if given for free?	1 (2.2)	4 (8.7)	41 (89.1)	0	1 (17.0)	5 (83.0)	1 (3.0)	3 (8.0)	36 (89.0)	0.52
Would you like to receive the HPV vaccine if you will have to pay for it?	4 (8.7)	3 (6.5)	39 (84.8)	1 (17.0)	0	5 (83.0)	3 (8.0)	3 (8.0)	34 (84.0)	0.65
Would you like your (future) daughters to receive the HPV vaccine if given for free?	1 (2.2)	3 (6.5)	42 (91.3)	0	1 (17.0)	5 (83.0)	1 (3.0)	2 (5.0)	37 (92.0)	0.44
Would you like your (future) daughters to receive the HPV vaccine if you will have to pay for it?	1 (2.2)	5 (10.8)	40 (87.0)	0	1 (17.0)	5 (83.0)	1 (3.0)	4 (10)	35 (87.0)	0.59

n, number of participants; WBJ, West Bank and Jerusalem; HPV, human papillomavirus

Data are presented as frequencies and percentages

## Discussion

The overall awareness of HPV and its vaccine in this study was extremely low with only 0.5% of the participants having good awareness. Participants from the WBJ demonstrated better awareness than participants from the Gaza Strip. Completing post-secondary education, being employed or a student, and having a higher monthly income were associated with an increase in the likelihood of having good awareness. Conversely, visiting governmental hospitals or primary healthcare centers was associated with a lower likelihood to display good awareness. A small proportion of women who had heard of HPV reported their familiarity with its vaccine. However, women from the WBJ were more likely than women from the Gaza Strip to have heard of the HPV vaccine. The majority of women in this study would agree to receive the HPV vaccine if it was given without cost as well as if they had to pay for it. In addition, most women would agree to their daughters receiving the HPV vaccine for free or at a cost to themselves.

Availability and uptake of the HPV vaccine could play a crucial role in the prevention and reduction of morbidity and mortality of CC [41, 42]. This study assessed the Palestinian women’s awareness level of HPV in addition to its vaccine and examined the acceptability of the HPV vaccine to provide baseline information for the implementation of future awareness campaigns and to facilitate the launch of HPV immunization programs. HPV immunization programs could be critical, especially with the lack of CC screening programs in low-income settings accompanied by high mortality as in Palestine [2].

### Good awareness of HPV and its vaccine and the associated factors

Effective implementation of HPV vaccination programs is considered one of the cornerstones to reducing the burden of CC, particularly in low- and middle-income countries [14, 41–43]. For a vaccination program to be effective, both awareness of its benefits and availability are important to ensure wide uptake among the population [43]. This study demonstrated low awareness of HPV and its vaccine among Palestinian women with only 0.5% of participants displaying good awareness. Similar results were found in studies from India, Australia, Ghana, Saudi Arabia, Lebanon, Jordan, Qatar, the United Arab Emirates, and Iraq [30, 44–48]. Although the World Health Organization stated in its CC prevention and control guidelines that the prevention and early detection of CC should be the main objective especially in low- and middle-income countries, more efforts are needed to achieve this [49–52]. Effective vaccination programs coupled with greater awareness facilitating public engagement with a vaccination program could lead to a significant reduction in CC morbidity and mortality in low- and middle-income countries [43].

A contributing factor to the low awareness of HPV in this study could be the neglect of speaking of HPV, particularly by healthcare workers who are considered a good source of shaping women's knowledge during their visits to healthcare facilities [18–20]. Moreover, the lack of freedom of movement impacts the Palestinians’ lives in the Gaza Strip, including health professionals’ participation in conferences and fellowships [53]. This might restrict health professionals’ exposure to the impact of HPV on CC and the effectiveness of the HPV vaccine, which might contribute to inadequate knowledge about it. This decreases the women’s chance of hearing about HPV and its vaccine in healthcare facilities. Therefore, low awareness of HPV and its vaccine could be expected, considering the lack of the HPV vaccination from the national immunization program alongside the absence of public education interventions regarding HPV in Palestine, as in other regional countries [48].

In line with other studies [30, 45–47], completing post-secondary education was associated with a higher likelihood of having a good awareness of HPV and its vaccine. This emphasizes the need to enrich school curricula with more information about health-related topics including HPV-related infection and its vaccine. In addition, targeting university curricula could be an efficient approach to promoting women’s awareness about HPV, its vaccine, and its relation to CC. Lambert and colleagues showed that a brief educational intervention increased college students’ performance on an HPV knowledge assessment from 45 to 79% after three months from the intervention [54]. Such educational interventions can also be tailored to address the concerns women have about the HPV vaccine to increase its acceptability, which ultimately will lead to the successful integration of HPV immunization into the national program [55].

In this study, being employed and having a higher monthly income were associated with an increase in the likelihood to display good awareness, which is in concordance with a previous study among Lebanese women [47]. Employed women might be more able to interact with well-educated colleagues, where they can talk about their own and relatives’ experiences including those related to health issues. In addition, women with higher monthly income might have better chances to access more health-related information through their visits to private healthcare facilities or other resources (e.g., searching the internet.)

Contrary to other studies that addressed CC awareness of symptoms and risk factors in Palestine, women recruited from healthcare centers were less likely to display good awareness of HPV and its vaccine [18, 19]. This could highlight that HPV is not well discussed in public discourse or health education in Palestine. In addition, religion and culture play a role in shaping a conservative attitude toward sexual behaviors as well as the popular practice of male circumcision in Palestine, which is considered a protective factor against HPV infection [56]. In addition, discussing sexual issues, including sexual health education, was shown to be relatively rare among people in Arab countries [48, 57, 58].

### Awareness level in the Gaza Strip versus the WBJ

Several factors might contribute to the finding that participants from the WBJ were more likely than participants from the Gaza Strip to have a good awareness level. Firstly, women in the Gaza Strip are limited in their ability to travel, even to get treatment, compared with women in the WBJ. Since 2006, Palestinians in the Gaza Strip have suffered from increased movement restrictions, making it very difficult to leave or enter the Gaza Strip [53, 59]. The ability to travel as well as the travel distance and time were shown to be associated with a change in the utilization of healthcare services [60, 61]. Access to healthcare and interaction with healthcare professionals could play a key role in shaping women’s health literacy [18–20, 37, 38]. Secondly, there is more digital deprivation in the Gaza Strip than in the WBJ. This is mainly because of the electricity crisis in the Gaza Strip, where power supplies are provided only for a few hours a day on a rolling blackout schedule affecting all aspects of daily life including the interest to surf the internet for health-related information [62, 63]. Finally, the lower socioeconomic status in the Gaza Strip than in the WBJ and the higher unemployment rates (49.1% in Gaza vs. and 14.8% in the WBJ) and greater poverty (64% in Gaza vs. 36% in the WBJ) could add another layer to this difference in awareness between the two areas [64–66] The decline in standards of living may have forced citizens in the Gaza Strip to think exclusively about fulfilling their daily basic needs, which could indirectly negatively impact their ability to read and educate themselves in health-related topics.

### Acceptability of HPV vaccine

Low knowledge about HPV infection and the vaccine could be a contributor to the poor acceptability of the HPV vaccine [47, 67]. In contrast to previous studies [68–70], participants in this study had poor knowledge of HPV and its vaccine. However, there was a positive attitude among Palestinian women, in both the WBJ and the Gaza Strip, towards HPV vaccination. The reason for this acceptance could be that the small proportion of participants who were aware of HPV and its vaccine had the knowledge about the protective role of the vaccine against HPV infection and its related cancers [68]. To date, the HPV vaccine is not provided as part of the national vaccination program in Palestine [16]. The findings in this study encourage considering the inclusion of the HPV vaccine in the program. This is supported by the lack of difference in acceptance of the HPV vaccine among women in the Gaza Strip versus the WBJ. This also suggests that any standardized intervention across Palestine to mitigate the barriers to receiving the HPV vaccine might have a homogenous impact among all Palestinian women. This study did not look at those barriers and further research is needed to identify them in order to formulate reasonable interventions to address them.

### Future directions

The findings of this study showed the substantial need to raise women’s awareness of HPV and its vaccine in Palestine. Enhancing school and university curricula with more in-depth information about health-related topics, including HPV infection and vaccine, should be considered. In addition, targeted educational interventions are needed to promote the knowledge of women of the reproductive age visiting governmental hospitals and primary healthcare centers. Moreover, educating healthcare professionals about the impact of HPV on CC and the effectiveness of its vaccine might be vital to achieving a successful launch of the HPV vaccine campaign in Palestine. There is a need to include the HPV vaccine in the Palestinian national vaccination program supported by the good acceptability among women demonstrated in this study [71].

### Strengths and limitations

The main strengths of this study include the large sample size and the inclusion of women from different places and governorates across Palestine to resemble the Palestinian community. In addition, the high response rate and the greater proportion of women included in their reproductive age showed a trend toward acceptance of such questionnaires by the target population. Furthermore, the low baseline knowledge about HPV and its vaccine in this study identifies an area for improvement to increase the chances of successful future inclusion of the HPV vaccine in the national vaccination program in Palestine.

Limitations of this study include convenience sampling, as it does not guarantee the generalizability of the findings. However, the large sample size, high response rate, and coverage of most geographical areas across Palestine may have mitigated this limitation. Another limitation could be that adolescents were not included, which could have provided an opportunity to explore their awareness and attitudes towards HPV and its vaccine. Nonetheless, assessing adolescents’ awareness may need a different study design (e.g., a qualitative study). Finally, the exclusion of participants with medical backgrounds might possibly have reduced the number of participants with good awareness. Nevertheless, their exclusion was intended to increase the relevancy of this study to measure public awareness.

## Conclusion

The overall awareness of women about HPV and its vaccine was extremely low with only 0.5% demonstrating good awareness. Factors associated with good awareness included living in the WBJ, completing post-secondary education, being employed or a student, and having a higher monthly income. Only 8.9% of women who had heard of HPV knew about its vaccine. A low awareness level of HPV and its vaccine could be expected considering the lack of the HPV vaccination in the national immunization program coupled with the absence of public education interventions. Most women showed good acceptance of the HPV vaccine, despite the vaccine not being part of the national vaccination program, which supports the necessary inclusion of the HPV vaccine in the national immunization schedule.

## Data Availability

The data analyzed during the study is available from the corresponding author upon reasonable request.

## Abbreviations

CC: Cervical cancer
HPV: Human papillomavirus
WBJ: West Bank and Jerusalem
MoH: Ministry of health
IQR: Interquartile range
CI: Confidence interval
OR: Odds ratio

## References

[1] Sung H, Ferlay J, Siegel RL, Laversanne M, Soerjomataram I, Jemal A (2021). Global cancer statistics 2020: GLOBOCAN estimates of incidence and mortality worldwide for 36 cancers in 185 countries. CA Cancer J Clin.

[2] Arbyn M, Weiderpass E, Bruni L, de Sanjosé S, Saraiya M, Ferlay J (2020). Estimates of incidence and mortality of cervical cancer in 2018: a worldwide analysis. Lancet Glob Health.

[3] World Health Organization. Cervical cancer: an NCD we can overcome 2018. https://bit.ly/3jQZ152. Accessed 30 Oct 2021.

[4] World Health Organization. Improving data for decision-making: a toolkit for cervical cancer prevention and control programmes. https://bit.ly/2RhPwAr. Accessed 30 Oct 2021.

[5] Wardak S (2016). Human papillomavirus (HPV) and cervical cancer. Med Dosw Mikrobiol.

[6] Okunade KS. Human papillomavirus and cervical cancer. J Obstet Gynaecol. 2019;1–7. 10.1080/01443615.2019.1674261 31825273

[7] de Martel C, Plummer M, Vignat J, Franceschi S (2017). Worldwide burden of cancer attributable to HPV by site, country and HPV type. Int J Cancer.

[8] Arbyn M, Xu L, Simoens C, Martin-Hirsch PP. Prophylactic vaccination against human papillomaviruses to prevent cervical cancer and its precursors. Cochrane Database Syst Rev. 2018;5(5):Cd009069. 10.1002/14651858.CD009069.pub3 PMC6494566 29740819

[9] Ali MAM, Bedair RN, Abd El Atti RM (2019). Cervical high-risk human papillomavirus infection among women residing in the Gulf Cooperation Council countries: prevalence, type-specific distribution, and correlation with cervical cytology. Cancer Cytopathol.

[10] Lubbad AM, Al-Hindi AI (2007). Bacterial, viral and fungal genital tract infections in Palestinian pregnant women in Gaza, Palestine. West Afr J Med.

[11] Simon AE, Wardle J, Grimmett C, Power E, Corker E, Menon U (2012). Ovarian and cervical cancer awareness: development of two validated measurement tools. J Fam Plann Reprod Health Care.

[12] Falcaro M, Castañon A, Ndlela B, Checchi M, Soldan K, Lopez-Bernal J (2021). The effects of the national HPV vaccination programme in England, UK, on cervical cancer and grade 3 cervical intraepithelial neoplasia incidence: a register-based observational study. Lancet.

[13] Burd EM (2003). Human papillomavirus and cervical cancer. Clin Microbiol Rev.

[14] Jit M, Brisson M, Portnoy A, Hutubessy R (2014). Cost-effectiveness of female human papillomavirus vaccination in 179 countries: a PRIME modelling study. Lancet Glob Health.

[15] Barra F, Della Corte L, Noberasco G, Foreste V, Riemma G, Di Filippo C (2020). Advances in therapeutic vaccines for treating human papillomavirus-related cervical intraepithelial neoplasia. J Obstet Gynaecol Res.

[16] Halahleh K, Gale RP (2018). Cancer care in the Palestinian territories. Lancet Oncol.

[17] Annual Health Report for Ministry of Health in Palestine 2020. https://bit.ly/3nHpymj. Accessed 30 Oct 2021.

[18] Elshami M, Al-Slaibi I, Abukmail H, Alser M, Radaydeh A, Alfuqaha A (2021). Knowledge of Palestinian women about cervical cancer warning signs: a national cross-sectional study. BMC Public Health.

[19] Elshami M, Thalji M, Abukmail H, Al-Slaibi I, Alser M, Radaydeh A (2021). Knowledge of cervical cancer risk factors among Palestinian women: a national cross-sectional study. BMC Womens Health.

[20] Elshami M, Yaseen A, Alser M, Al-Slaibi I, Jabr H, Ubaiat S (2021). Knowledge of ovarian cancer symptoms among women in Palestine: a national cross-sectional study. BMC Public Health.

[21] Marlow LA, Zimet GD, McCaffery KJ, Ostini R, Waller J (2013). Knowledge of human papillomavirus (HPV) and HPV vaccination: an international comparison. Vaccine.

[22] Jradi H, Bawazir A (2019). Knowledge, attitudes, and practices among Saudi women regarding cervical cancer, human papillomavirus (HPV) and corresponding vaccine. Vaccine.

[23] Bardají A, Mindu C, Augusto OJ, Casellas A, Cambaco O, Simbine E (2018). Awareness of cervical cancer and willingness to be vaccinated against human papillomavirus in Mozambican adolescent girls. Papillomavirus Res.

[24] Farazi PA, Siahpush M, Michaud TL, Kim J, Muchena C (2019). Awareness of HPV and cervical cancer prevention among university health sciences students in cyprus. J Cancer Educ.

[25] Wheldon CW, Krakow M, Thompson EL, Moser RP (2019). National trends in human papillomavirus awareness and knowledge of human papillomavirus-related cancers. Am J Prev Med.

[26] Baloch Z, Yasmeen N, Li Y, Zhang W, Lu H, Wu X (2017). Knowledge and awareness of cervical cancer, human papillomavirus (HPV), and HPV vaccine among HPV-infected Chinese women. Med Sci Monit.

[27] Ortashi O, Raheel H, Shalal M, Osman N (2013). Awareness and knowledge about human papillomavirus infection and vaccination among women in UAE. Asian Pac J Cancer Prev.

[28] Gamaoun R (2018). Awareness and knowledge about cervical cancer prevention methods among Tunisian women. J Prev Med Hyg.

[29] Mouallif M, Bowyer HL, Festali S, Albert A, Filali-Zegzouti Y, Guenin S (2014). Cervical cancer and HPV: awareness and vaccine acceptability among parents in Morocco. Vaccine.

[30] Rashid S, Labani S, Das BC (2016). Knowledge, awareness and attitude on HPV, HPV vaccine and cervical cancer among the college students in India. PLoS ONE.

[31] Rajiah K, Maharajan MK, Chin NS, Num KS (2015). Awareness and acceptance of human papillomavirus vaccination among health sciences students in Malaysia. Virusdisease.

[32] Yörük S, Açıkgöz A, Ergör G (2016). Determination of knowledge levels, attitude and behaviors of female university students concerning cervical cancer, human papiloma virus and its vaccine. BMC Womens Health.

[33] Centers for Disease Control and Prevention. Basic information about HPV and cancer. https://bit.ly/2ZbvagA. Accessed 20 Nov 2021.

[34] Centers for Disease Control and Prevention. HPV vaccine recommendations. https://bit.ly/30GHsyp. Accessed 20 Nov 2021.

[35] Harvard Humanitarian Initiative. KoBoToolbox. https://www.kobotoolbox.org. Accessed 30 Oct 2021.

[36] Palestinian Central Bureau of Statistics: On the occasion of the International Workers' Day, H.E. Dr. Ola Awad, President of PCBS, presents the current status of the Palestinian labour force. https://bit.ly/3QqhenP. Accessed 30 July 2022.

[37] Elshami M, Elshami A, Alshorbassi N, Alkhatib M, Ismail I, Abu-Nemer K (2020). Knowledge level of cancer symptoms and risk factors in the Gaza Strip: a cross-sectional study. BMC Public Health.

[38] Elshami M, Bottcher B, Alkhatib M, Ismail I, Abu-Nemer K, Hana M (2021). Perceived barriers to seeking cancer care in the Gaza Strip: a cross-sectional study. BMC Health Serv Res.

[39] Elshami MTM, AbuKmail H, et al. Knowledge of cervical cancer risk factors among Palestinian women: a national cross-sectional study. BMC Women’s Health. 2021. 10.1186/s12905-021-01510-2 PMC8561913 34727914

[40] Elshami MYA. Knowledge of overian cancer symptoms among women in Palestine: a national cross-sectional study. BMC Public Health. 2021. 10.1186/s12889-021-12044-5 PMC8567700 34732142

[41] World Health Organization. Guide to cancer early diagnosis 2017. https://bit.ly/3Cuvw0b. Accessed 30 Oct 2021.

[42] Taghizadeh Asl R, Van Osch L, De Vries N, Zendehdel K, Shams M, Zarei F (2020). The role of knowledge, risk perceptions, and cues to action among Iranian women concerning cervical cancer and screening: a qualitative exploration. BMC Public Health.

[43] Poirier B, Sethi S, Garvey G, Hedges J, Canfell K, Smith M (2021). HPV vaccine: uptake and understanding among global Indigenous communities: a qualitative systematic review. BMC Public Health.

[44] Netfa F, King C, Davies C, Rashid H, Tashani M, Booy R (2021). Knowledge, attitudes, and perceptions of the Arabic-speaking community in Sydney, Australia, toward the human papillomavirus (HPV) vaccination program: a qualitative study. Vaccines (Basel).

[45] Williams MS, Kenu E, Adanu A, Yalley RA, Lawoe NK, Dotse AS (2019). Awareness and beliefs about cervical cancer, the HPV vaccine, and cervical cancer screening among Ghanaian women with diverse education levels. J Cancer Educ.

[46] Hussain AN, Alkhenizan A, McWalter P, Qazi N, Alshmassi A, Farooqi S (2016). Attitudes and perceptions towards HPV vaccination among young women in Saudi Arabia. J Family Community Med.

[47] Abou El-Ola MJ, Rajab MA, Abdallah DI, Fawaz IA, Awad LS, Tamim HM (2018). Low rate of human papillomavirus vaccination among schoolgirls in Lebanon: barriers to vaccination with a focus on mothers' knowledge about available vaccines. Ther Clin Risk Manag.

[48] Alsous MM, Ali AA, Al-Azzam SI, Abdel Jalil MH, Al-Obaidi HJ, Al-Abbadi EI (2021). Knowledge and awareness about human papillomavirus infection and its vaccination among women in Arab communities. Sci Rep.

[49] WHO Guidelines Approved by the Guidelines Review Committee. Comprehensive cervical cancer control: a guide to essential practice. Geneva: World Health Organization Copyright © World Health Organization 2014; 2014. 25642554

[50] Esteva M, Leiva A, Ramos M, Pita-Fernández S, González-Luján L, Casamitjana M (2013). Factors related with symptom duration until diagnosis and treatment of symptomatic colorectal cancer. BMC Cancer.

[51] Macdonald S, Macleod U, Campbell NC, Weller D, Mitchell E (2006). Systematic review of factors influencing patient and practitioner delay in diagnosis of upper gastrointestinal cancer. Br J Cancer.

[52] Simon AE, Waller J, Robb K, Wardle J (2010). Patient delay in presentation of possible cancer symptoms: the contribution of knowledge and attitudes in a population sample from the United kingdom. Cancer Epidemiol Biomark Prev.

[53] Al JAZEERA. Report: Gaza travel remains difficult. https://bit.ly/3bsgqw9. Accessed 30 Oct 2021.

[54] Lambert EC (2001). College students' knowledge of human papillomavirus and effectiveness of a brief educational intervention. J Am Board Fam Pract.

[55] Control ECfDPa. Introduction of HPV vaccines in European Union countries: an update 2012.

[56] Larke N, Thomas SL, Dos Santos SI, Weiss HA (2011). Male circumcision and human papillomavirus infection in men: a systematic review and meta-analysis. J Infect Dis.

[57] Alomair N, Alageel S, Davies N, Bailey JV (2020). Factors influencing sexual and reproductive health of Muslim women: a systematic review. Reprod Health.

[58] Gamaoun R (2018). Knowledge, awareness and acceptability of anti-HPV vaccine in the Arab states of the Middle East and North Africa Region: a systematic review. East Mediterr Health J.

[59] Join HuffPost. 10 Things Palestinians can’t do because of the Israeli occupation 2016. https://bit.ly/3GE9SJ8. Accessed 30 Oct 2021.

[60] Oh JH (2019). Educational expansion and health disparities in Ethiopia, 2005–2016. Soc Sci Med.

[61] Kohler RE, Lee CN, Gopal S, Reeve BB, Weiner BJ, Wheeler SB (2015). Developing a discrete choice experiment in Malawi: eliciting preferences for breast cancer early detection services. Patient Prefer Adherence.

[62] United Nations Office for the Coordination of Humanitarian Affairs. The impact of the electricity crisis on the humanitarian and living conditions in the Gaza Strip-survey study, November 2020. https://bit.ly/3CJFeMt. Accesssed 30 Oct 2021.

[63] Office for the Coordination of Humanitarian Affairs. The humanitarian impact of Gaza's electricity and fuel crises 2015. https://bit.ly/3jQz5GS. Accessed 30 Oct 2021.

[64] Economic and Social Commission for Western Asia. Monitoring socio-economic conditions in the occupied Palestinian territories: macroeconomics benchmarking and trend analysis. 2017. https://bit.ly/3vZAZtj. Accessed 20 Nov 2021.

[65] United Nations Office for the Coordination of Humanitarian Affairs. Gaza unemployment rate in the second quarter of 2020: 49.1%. https://bit.ly/3HQXhDk. Accessed 20 Nov 2021..

[66] Unitied Nations. Israeli occupation of Palestinian territory: in facts and figures. https://bit.ly/3kSwGLZ. Accessed 20 Nov 2021.

[67] Fu LY, Bonhomme LA, Cooper SC, Joseph JG, Zimet GD (2014). Educational interventions to increase HPV vaccination acceptance: a systematic review. Vaccine.

[68] He J, He L (2018). Knowledge of HPV and acceptability of HPV vaccine among women in western China: a cross-sectional survey. BMC Womens Health.

[69] López N, Garcés-Sánchez M, Panizo MB, de la Cueva IS, Artés MT, Ramos B (2020). HPV knowledge and vaccine acceptance among European adolescents and their parents: a systematic literature review. Public Health Rev.

[70] Ezat SW, Hod R, Mustafa J, Mohd Dali AZ, Sulaiman AS, Azman A (2013). National HPV immunisation programme: knowledge and acceptance of mothers attending an obstetrics clinic at a teaching hospital, Kuala Lumpur. Asian Pac J Cancer Prev.

[71] Wilailak S, Kengsakul M, Kehoe S (2021). Worldwide initiatives to eliminate cervical cancer. Int J Gynaecol Obstet.

